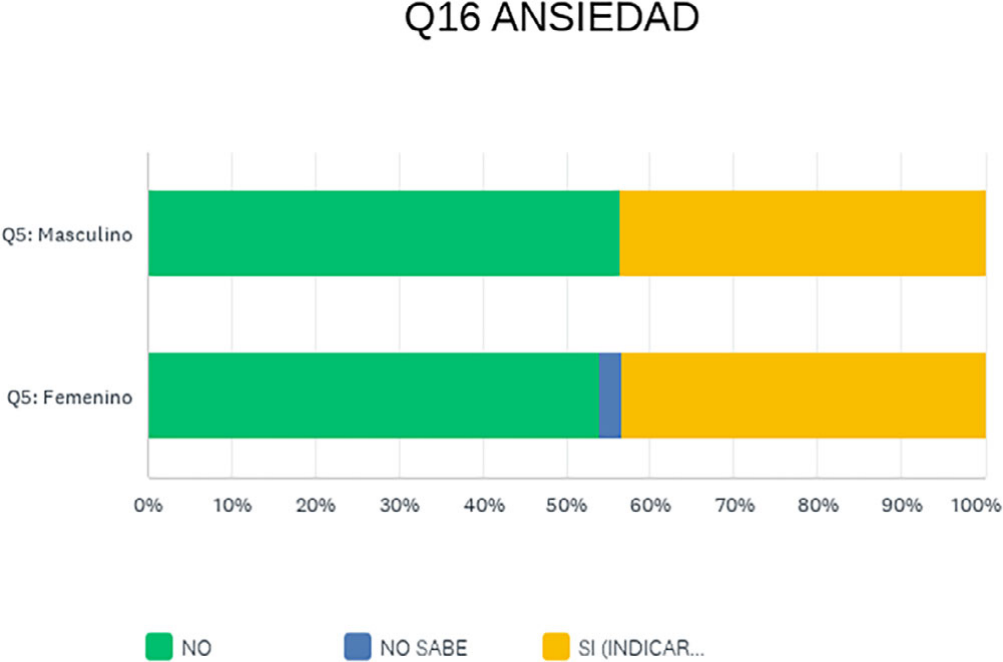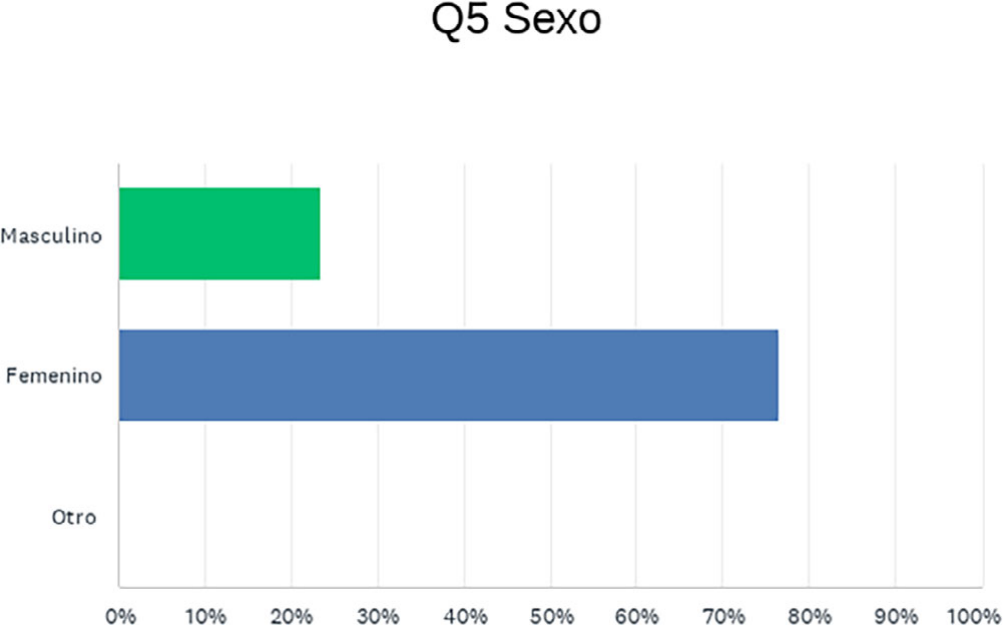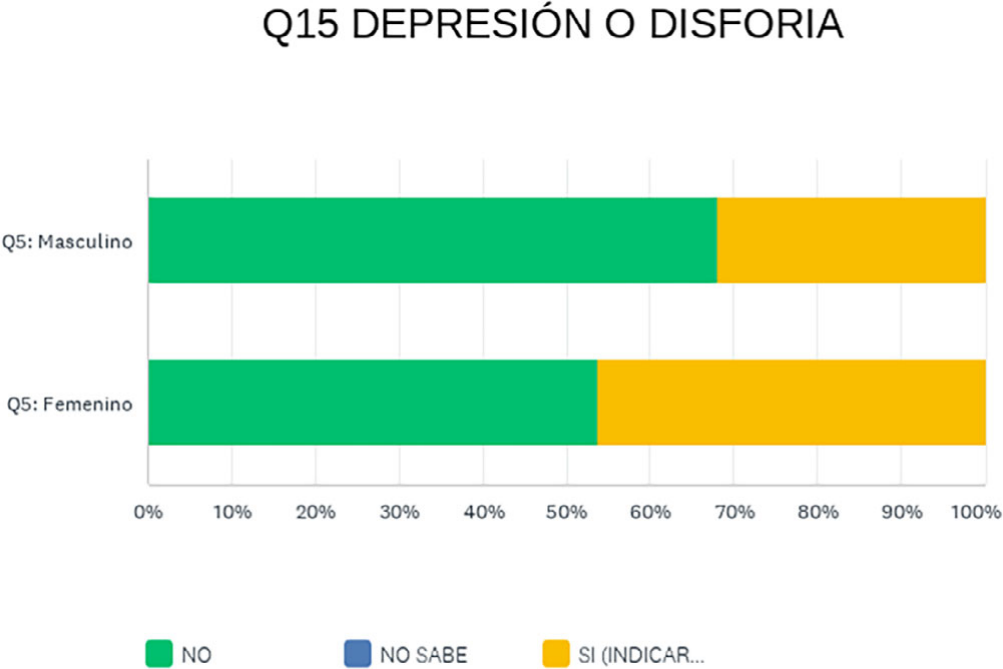# Cross‐sectional study of the impact of psychosocial factors on cognitive and behavioral symptoms in patients at the University Hospital of Buenos Aires

**DOI:** 10.1002/alz.091924

**Published:** 2025-01-09

**Authors:** Cynthia Dunovits, Luis Ignacio Brusco, Natividad Olivar, Sandra Germani

**Affiliations:** ^1^ ALZAR, Argentine Alzheimer s Association, Buenos Aires, Buenos AIRES Argentina; ^2^ University of Buenos Aires, Buenos Aires Argentina; ^3^ ALZAR ‐ Argentine Alzheimer’s Association, Buenos Aires, CABA Argentina; ^4^ National Scientific and Technical Research Council (CONICET), Buenos Aires Argentina; ^5^ Universidad de Buenos Aires, Buenos Aires Argentina; ^6^ ALZAR ‐ Argentine Alzheimer’s Association, CABA, Buenos Aires Argentina

## Abstract

**Background:**

Increasing evidence points to the possible risk roles of psychosocial factors (lack of education, active social participation, physical exercise and mentally stimulating activity, economic instability, traumatic life events) in the pathogenic process and clinical manifestation of dementia disorders.

In recent years in our country, in the context of a complex inflationary process, there has been an increase in all indicators of vulnerability and poverty, exceeding 40% of the population below the poverty line in 2023 and around 9% below the indigence line.

**Method:**

In the context of International Alzheimer’s Day, the Alzheimer’s Disease Awareness Week was held at the Hospital de Clínicas of Buenos Aires. In it, spontaneous attention was provided to 100 patients between 60 and 85 years old with cognitive complaints, evaluating education, occupation, socio‐environmental variables along with anxiety scales (GAD); affective (PHQ), minimental and NPI.

**Result:**

In our sample of one hundred patients, a clear prevalence of women in the consultation with a medium educational level was observed. An increase in the incidence of anxious symptoms (40%) and depressive symptoms (40%) was evident; followed by irritability (30%); sleep disturbances (35%); apathy (20%).

**Conclusion:**

Psychosocial factors play a fundamental role in the incidence, onset and progression of cognitive conditions, which is why they should be a priority in dementia prevention policies. The prevalence of women’s consultations and their earlier access in a preventive modality also shows their more fluid contact and circulation in the health system and particularly in primary care.